# Weibel-Palade body exocytosis as a therapeutic target to improve hemodynamics in Gram-positive sepsis

**DOI:** 10.1186/cc11718

**Published:** 2012-11-14

**Authors:** JY Lee, HM Linge, K Ochani, YZ Zhang, EJ Miller

**Affiliations:** 1 The Elmezzi Graduate School of Molecular Medicine, Manhasset, NY, USA; 2 The Feinstein Institute for Medical Research, Heart and Lung Research Center, Manhasset, NY, USA; 3 Hofstra University School of Medicine, Heart and Lung Research Center, Manhasset, NY, USA

## Background

Gram-positive bacterial lung infection is commonly associated with sepsis, and particularly important in older individuals. Systemic vascular dysfunction associated with sepsis is characterized by vascular permeability that can lead to tissue hypoxia. Our studies focus on angiopoietin-2 (Ang-2), whose increased plasma concentration is associated with severity of lung injury and with mortality. Ang-2 is stored in the Weibel-Palade body (WPB), an endothelial-specific secretory organelle. Here, we examine the release of Ang-2 from primary human pulmonary microvascular endothelial cells (HPMECs) *in vitro *stimulated with Gram-positive bacterial cell wall components. We then used our model of pulmonary infection to investigate the use of an inhibitor of WPB exocytosis to prevent systemic inflammation and hemodynamic instability.

## Methods

HPMECs were treated with lipoteichoic acid (LTA; 50 to 100 μg/ml) and peptidoglycan (PGN; 167 to 333 μg/ml) in the presence or absence of TAT-NSF700 fusion peptide (10 μM) to inhibit WPB exocytosis. Ang-2 in culture medium was measured by ELISA, and WPB exocytosis assessed using immunofluorescent (IF) staining of Ang-2. HPMEC monolayer permeability was measured using FITC-dextran. Male C57/Bl6 mice, 8 to 10 weeks old (*n *= 6/group), were pretreated with TAT-NSF700 (0.5 mg/kg) or saline intraperitoneally. Thirty minutes later, LTA (150 μg) and PGN (500 μg) or saline alone were instilled intratracheally. Pulse oximetry was assessed in awake mice prior to and 6 hours post instillation. The mice were then euthanized by exsanguination under anesthesia and bronchoalveolar lavage (BAL) was performed. BAL fluid total and differential counts were evaluated and protein and cytokine concentrations in plasma and BAL were assessed using commercial assays.

## Results

LTA-PGN increased Ang-2 levels dose dependently (up to ninefold, *P *= 0.003) in HPMEC culture medium within 30 minutes, which was blocked by TAT-NSF700 pretreatment. IF staining showed aggregation and localization of Ang-2 in the cytoplasm suggesting WPB exocytosis within 30 minutes. LTA-PGN also induced HPMEC permeability. LTA-PGN induced both local and systemic inflammation resulting in decreased heart and breath rates and oxygen saturation (94% vs. 97%, *P *= 0.038) at 6 hours. These physiological changes were prevented in mice pretreated with TAT-NSF700.

## Conclusion

LTA-PGN induced rapid Ang-2 secretion via WPB exocytosis and this release is associated with significant changes in permeability. *In vivo*, LTA-PGN induces significant changes in heart and breath rates and oxygen saturation that was prevented by inhibition of WPB exocytosis. Thus, we have defined WPB, a storage organelle for multiple proinflammatory mediators, as a potential target to control overwhelming inflammation in Gram-positive sepsis and improve tissue oxygenation and hemodynamics.

